# Examining pathology of Alzheimer’s disease via clusterin and Aβ interaction

**DOI:** 10.1002/alz.087898

**Published:** 2025-01-03

**Authors:** Danyeong Kim, Kyu Hwan Shim, Yunseo Gong, Hyewon Yang, Min Ju Kang, Seong Soo An

**Affiliations:** ^1^ Veterans Health Service Medical Center, Seoul Korea, Republic of (South); ^2^ Department of Bionano Technology, Gachon University, Seongnam Korea, Republic of (South)

## Abstract

**Background:**

Clusterin, a multifunctional glycoprotein, is implicated in Alzheimer’s disease (AD) pathogenesis due to its roles in Aβ aggregation and clearance. Hence, understanding the specific interactions between Clusterin and Aβ would be a crucial for unraveling AD mechanisms and exploring therapeutic avenues. Previous study reported that clusterin bound with Aβ directly. This study investigated the specific fractions of clusterin interacting with Aβ in AD. Our focus extended to analyzing patient samples, offering clinical relevance. This comprehensive approach aimed to deepen insights into AD pathology for potential therapeutic and diagnostic applications.

**Method:**

Clusterin was synthesized into 4 fragments (CLU #1, 2, 3, 4) and identified in Aβ fibrilization and oligomerization inhibition effects through thioflavin T (ThT) and Multiple detection system (MDS) assay. The fragments were conducted for Aβ inhibition effects through ThT and MDS assay. SH‐SY5Y cells were measured for Aβ protections of clusterin fragments. ELISA was assessed for clusterin and co‐interaction of clusterin‐Aβ in AD plasma samples.

**Result:**

Our results proved that clusterin inhibited Aβ fibrilization. Especially, clusterin #2 significantly decreased both Aβ fibrilization and oligomerization. The clusterin #3 also showed Aβ oligomerization inhibition effect, but less than the #2. The #2 and #3 increased cell viability after treatment of Aβ protofibrils. By extension to inhibit clusterin and Aβ from clinical samples, clusterin levels slightly decreased in AD plasma samples in comparison to healthy control. Next, co‐interactions of clusterin‐Aβ were decreased in AD samples.

**Conclusion:**

This study highlighted the inhibition effect of Aβ by clusterin through its close interactions. Especially, specific fragments (#2) of clusterin strongly decreased Aβ oligomerization, and recovered cell viability after Aβ protofibril treatment. This suggested that clusterin supported the direct interaction with Aβ, furthermore involving its removal. In clinical aspects, the decreased levels of clusterin in AD plasma were observed, along with reduced clusterin‐Aβ co‐interactions, which indicated the potential impairments in Aβ clearance mechanisms, contributing to AD pathology progression. The findings contributed to our understanding of the complex interplays between Clusterin and Aβ in AD, offering insights for therapeutic and diagnostic development.

